# Probabilistic Approach to COVID-19 Data Analysis and Forecasting Future Outbreaks Using a Multi-Layer Perceptron Neural Network

**DOI:** 10.3390/diagnostics12102539

**Published:** 2022-10-19

**Authors:** Riaz Ullah Khan, Sultan Almakdi, Mohammed Alshehri, Rajesh Kumar, Ikram Ali, Sardar Muhammad Hussain, Amin Ul Haq, Inayat Khan, Aman Ullah, Muhammad Irfan Uddin

**Affiliations:** 1 Yangtze Delta Region Institute (Huzhou), University of Electronic Science and Technology of China, Huzhou 313001, China; 2 Department of Computer Science, College of Computer Science and Information Systems, Najran University, Najran 55461, Saudi Arabia; 3 School of Automation Engineering, University of Electronic Science and Technology of China, Chengdu 611731, China; 4 Department of Mathematical Sciences, Balochistan University of Information Technology, Engineering and Management Sciences, Quetta 87300, Pakistan; 5 School of Computer Science and Engineering, University of Electronic Science and Technology of China, Chengdu 611731, China; 6 Department of Computer Science, University of Buner, Buner 19290, Pakistan; 7 Institute of Computer Science, Kohat University of Science and Technology, Kohat 26000, Pakistan

**Keywords:** COVID-19 variants, artificial neural network, B.1.1.529, Omicron, forecasting

## Abstract

The present outbreak of COVID-19 is a worldwide calamity for healthcare infrastructures. On a daily basis, a fresh batch of perplexing datasets on the numbers of positive and negative cases, individuals admitted to hospitals, mortality, hospital beds occupied, ventilation shortages, and so on is published. Infections have risen sharply in recent weeks, corresponding with the discovery of a new variant from South Africa (B.1.1.529 also known as Omicron). The early detection of dangerous situations and forecasting techniques is important to prevent the spread of disease and restart economic activities quickly and safely. In this paper, we used weekly mobility data to analyze the current situation in countries worldwide. A methodology for the statistical analysis of the current situation as well as for forecasting future outbreaks is presented in this paper in terms of deaths caused by COVID-19. Our method is evaluated with a multi-layer perceptron neural network (MLPNN), which is a deep learning model, to develop a predictive framework. Furthermore, the Case Fatality Ratio (CFR), Cronbach’s alpha, and other metrics were computed to analyze the performance of the forecasting. The MLPNN is shown to have the best outcomes in forecasting the statistics for infected patients and deaths in selected regions. This research also provides an in-depth analysis of the emerging COVID-19 variants, challenges, and issues that must be addressed in order to prevent future outbreaks.

## 1. Introduction

The present COVID-19 outbreak is a serious global crisis for healthcare infrastructures. The pandemic has triggered a crisis due to which, schools, administrative institutions, and financial institutions such as banks have been shut down in many major countries. Notably, such disruptions not only cause problems for people in the short term but also have long-term effects, for example, an increase in unemployment [1]. According to a study [2], the situation causes a 2.5–3% decline in the economic stability of GDPs globally every month. Furthermore, based on previous crises, it appears that younger and less-educated workers are the most financially impacted [2]. COVID-19 is thought to have originated from animals. If communities do not follow preventive policies for this highly contagious disease, COVID-19 can spread easily to healthy humans through close contact. Traveling has been the main cause of the huge spread [3,4]. In the early days of the COVID-19 pandemic, almost all reported cases were symptomatic. In a study by Noh J and Danuser G [5] of 50 countries, the number of actual COVID-19 patients in 25 of those countries was predicted to be 5 to 20 times larger than confirmed infected cases. In several European countries in March 2020, the number of total cases/infected patients was around 2.5 times higher than actual reported patients, and currently, it is estimated that the number of unseen infected cases is still 1.5 times higher than reported cases because undiscovered or unseen patients could be symptom-less or exhibit very subtle illness symptoms [6]. Researchers in the fields of pharmacy, chemistry, mathematics, physics, statistics, economics, computer science, geophysics, and medicine have joined hands to fight against COVID-19. However, no one has reached a firm conclusion yet on how to overcome this problem. Furthermore, the structure and symptoms are always mutating. The flu, body temperature, coughing, and shortness of breath are the initial indications of the COVID-19 virus. The severe side effects of this infection may cause acute respiratory disorder (a severe form of asthma), pneumonia, heart failure, renal failure, and possibly death in the subsequent stages [7]. The COVID-19 spread could be significantly slowed down by employing precautionary measures such as minimizing direct contact, social isolation, and smart lockdowns [8].

The accurate and robust forecasting of COVID-19 cases and deaths can assist government interventions and encourage the general public to consider effective actions to slow down the spread of this disease [9]. Researchers have conducted multiple studies to explore the COVID-19 associated risk factors and emotional effects, covering various categories such as nature, health, lockdown, etc., using different models [8,10]. Machine learning models, such as random forest, support vector machine (SVM), K- nearest neighbors (KNN), artificial neural networks, and many others, have also been used to predict the COVID-19 situation [11]. The reproduction rate of a disease is of great concern to epidemiologists as this is what determines a pandemic; a reproduction rate greater than one indicates a pandemic in the population [12]. The nature of COVID-19 has been studied by taking a variety of mathematical models into account. The most used model for analyzing disease dynamics is the Susceptible-Infectious-Recovered (SIR) model. This model uses a system of differential equations that are time-dependent to predict epidemic growth. Researchers have extensively employed the SIR model and different modified forms to study Ebola and AIDS [13,14]. Godio et al. [15] studied the recent SARS-CoV-2 pandemic outbreak by taking data from Italy using the SEIR epidemiological model. They used a Particle Swarm Optimization (PSO) solver to create a stochastic method to fit the model parameters, which improved the predictability of the prediction in a medium run of thirty days. Their findings matched Spanish and South Korean statistics and forecasts. Baleanu et al. [16] and some other researchers [17,18] used the Caputo–Fabrizio derivative to create a COVID-19 fractional differential equation model. The data on COVID-19 reflect a sequence of observations and time-series prediction approaches e.g., artificial neural network-based methods and meta-predictors are all native to the statistics [19,20]. For time-series forecasting, ANNs are frequently used [21]. ANN-based techniques have many advantages over machine learning techniques and one of the key advantages is that ANN can be fed raw data and discover the desired features automatically [22]. ANNs give accurate results based on numerous factors such as performance, accuracy, latency, speed, convergence, and size [23,24]. It is important to note that this research relies on artificial neural networks (ANNs) for forecasting the COVID-19 situation in certain countries.

In this paper, we propose a model to forecast future COVID-19 scenarios in major countries and provide insights for government bodies and policymakers. This work also provides a detailed look at the current COVID-19 variants, challenges, and guidelines for preventing the outbreak effectively. This forecasting is intended to assist organizations, legislators, and the general public in implementing new tactics and reinforcing ongoing COVID-19 precautionary actions. Additionally, this study could aid in relieving the socioeconomic and psychological distress caused by COVID-19. The key contributions of this study are given below.

### Key Contributions

- Awareness about emerging variants of COVID-19: We have collected information about COVID-19 including its types and emerging variants. It is important to note that some of the variants can appear without any prior symptoms.
- Literature review: This article gives a brief overview of the related work recently undertaken in the field of COVID-19 forecasting using data mining approaches including machine learning, and deep learning techniques.
- Proposed Methodology: We proposed an artificial neural network-based methodology for the statistical analysis of the current pandemic situation in some eastern and western countries. The results show that our approach works well in terms of precision and model fitting to statistical data.
- Challenges and future directions: We discussed the current issues associated with utilizing Artificial Intelligence methods to resolve the COVID-19 pandemic. Furthermore, we demonstrate how machine learning and deep learning can assist in preventing the spread of COVID-19 in the future. We also address the potential future contributions of AI and blockchain-based solutions to analyze the outbreak response.

## 2. Coronavirus

Coronaviruses are indeed a huge family of viruses that are found both in humans and animals [25]. Seven different types have been identified, including the ones that caused COVID-19 and the SARS and MERS illnesses. According to initial estimations, the retrovirus seemed to be more contagious than the one that caused SARS, although it appeared to be less probable to provoke catastrophic illnesses. We still have a lot to learn about the novel coronavirus (COVID-19) [26].

### 2.1. Symptoms of COVID-19

COVID-19 has been related to a variety of indications, ranging from simple headaches to life-threatening diseases. Upon being exposed to the illness, symptoms and signs may appear after 2 to 14 days [27]. The severity of the symptoms varies from mild to severe. COVID-19 is a virus that can cause the following symptoms in patients:

- Temperature or chills
- Runny nose
- Coughing
- Breathing problems
- Fatigue
- Aches in the muscles or throughout the body
- Loss of smell or taste
- Diarrhea
- Sore throat
- Nausea or vomiting

This is not an extensive list of all symptoms and manifestation. The CDC [27] continues to update the list of possible symptoms whenever new information becomes available from research labs or other academic sources. COVID-19 infection appears to put elderly persons with serious medical conditions, such as diabetes, heart disease, or respiratory problems, at an increased risk of developing more serious conditions.

### 2.2. Types of Coronavirus

In a new study on COVID-19, UK-based scientists discovered that there are six different varieties of COVID-19 infection, each with its own set of symptoms.

1. **Flu-like without a temperature**
  Fatigue, muscle aches, absence of smell, sore throat, coughing, shortness of breath, and no temperature are some of the additional symptoms.
2. **Flu-like with temperature**
  Fatigue, absence of smell, sore throat, coughing, uncontrollable shaking, a decrease in hunger, and a temperature.
3. **Gastrointestinal**
  Fatigue, absence of smell, sore throat, a decrease in hunger, chest pain, no coughing, and diarrhea.
4. **Extreme level one, severe exhaustion**
  Fatigue, loss of smell, cough, chest pain, a temperature, and hoarseness.
5. **Extreme level two, misconception (uncertainty)**
  Fatigue, absence of smell, a decrease in hunger, coughing, sore throat, chest pain, a temperature, hoarseness, muscle pain, and confusion.
6. **Extreme level three, abdominal and pulmonary**
  Fatigue, absence of smell, a decrease in hunger, coughing, sore throat, chest pain, a temperature, hoarseness, and muscle pain.

### 2.3. Emerging Variants of COVID-19

New variants are emerging with time. For example, recently, a new mutant (B.1.1.529 also known as Omicron) has emerged, which is fast spreading and can pose a big threat to the effectiveness of COVID-19 vaccinations [28]. Researchers are closely monitoring this novel mutant of COVID-19. This variant contains various changes, which were earlier reported in other mutants, particularly Delta. This new variant has been observed to be expanding rapidly within South Africa. Nowadays, the main goal is to focus on its expansion. The said mutation was identified in Botswana on 11 November 2021 [29] and was identified in a South African traveler who traveled to Hong Kong. Omicron was added to the list of “variants of concern” by the WHO, which also contains Alpha, Beta, Gamma, and Delta. Viruses transform themselves all the time and the majority of mutations are minor. Some of these mutations may be harmful to the virus itself, whereas others can make the infection more aggressive or dangerous. Table 1 illustrates the alterations with the highest risk, which are described as the “variants of concern” and are regularly observed by healthcare practitioners. Regarding vaccinations against COVID-19, the vaccinations from Chinese Sinopharm, Pfizer, and AstraZeneca are very efficacious against the variations after two doses, whereas resistance after one dosage appears to be diminished [30].

There are several variants of SARS-CoV-2, including a brand-new, extremely contagious variant that was detected in the United Kingdom [26]. Another of these new variants is known as VOC202101/02 or P.1 and was reported in visitors from Brazil who traveled to Japan in January 2021. This gene contains the 1–4 nt insertion, three reductions, four identical modifications, and 17 distinct amino acid modifications [31]. Travel restrictions were implemented in an effort to stop the spread of P.1 throughout the nation after it was discovered in the United Kingdom [32]. However, another variety from Brazil (known in the UK as VUI202101/01) was discovered in the UK and comprises a minor recessive mutation. Eight instances of this type, which appeared to be of minimal significance, had been reported as of 14 January 2021. The “expansion and importance of this mutation continues under investigative process”, according to Public Health England (PHE). At same time as the English variant, the South African variant appeared and has since been found in at least 20 countries. According to South African genomic data, the 501Y.V2 mutation swiftly supplanted other circulating progenitors in the country because it appeared to have a greater infection rate and hence is more transmittable. The N501Y and E484K spike protein variants are present in this version, as they are in the English and Brazilian variants.

### 2.4. Variants of Interest (VOI)

There is significant proof that the differences in the variants have a massive effect on infectivity, disease intensity, and/or resistance, affecting the epidemiologic scenario in the EU/EEA [30]. There is at least reasonable certainty in the findings for these features, which included genetic, epidemiologic, and in vitro investigations. Additionally, all of the prerequisites for the variants of concern and under investigation listed in Table 2 apply. The indications are labeled to show whether they come from the variants themselves (v) or from mutations linked to the variants (m). Evidence with a “low confidence” rating is labeled to highlight that it is inconclusive. Blank fields or null fields indicate that there are no existing evaluations or scientific evidence for the category, whereas “no” means that there has been no change associated with the feature. B.1 is the comparable virus that is presumed to be “wild-type” (with D614G and no other spike protein modifications) [27].

### 2.5. Variants under Observation

SARS-CoV-2 variants under observation were discovered as indications through outbreak intelligence, rules-based genomic variant screening, and initial technical data [38]. There is some indication that they are similar to the VOIs in terms of quality; however, the evidence is either inadequate or is still to be examined by the ECDC [27]. One or more outbreaks in communities or proof of the communal spread of the mutation elsewhere in the world must have been established for the mutations mentioned in Table 3.

## 3. Related Work

Machine learning algorithms often employ data sequences collected over time as the input data to forecast the COVID-19 pandemic situation. The COVID-19 spread has been predicted using a variety of methodologies. The Long Short-Term Memory (LSTM) algorithm is one of the methodologies that has been used. The multi-layer perceptron (MLP), for example, is now being used to forecast the spread of COVID-19. This strategy has made it easier to anticipate the maximum number of COVID-19 victims, the highest proportion of survivors, and the highest number of fatalities per region in a specific time period [44].

Al-Qanes et al. [45] developed a more advanced form of the adaptive neuro-fuzzy infererence system (ANFIS) to calculate the infected patients in different four countries: United States, Iran, Italy, and Korea. Their approach was founded on the marine predators algorithm, a revolutionary nature-inspired optimization. The ANFIS variables were optimized using this technique, improving prediction accuracy. The model has shown efficient prediction performance for MAE, RMSE, MAPE, and $R^{2}$ [45]. Other research used an improved ANFIS model by integrating the flower pollination algorithm (FPA) and salp swarm algorithm (SSA). The proposed FPASSA-ANFIS framework was evaluated by employing verified data obtained from the WHO website. Additionally, the proposed model’s performance was evaluated using two different datasets of weekly infected patients [20].

The Susceptible-Exposed-Infectious-Recovered (SEIR) approach was used by Alsayed et al. [46] to forecast pandemic peaks in Malaysia. Researchers have utilized the ANFIS approach to anticipate the number of infected people in the short term. Additionally, researchers have hypothesized that extending the treatment time may lessen the severity of the pandemic at its height. The MAPE, RMSE, and $R^{2}$ values for this study were 2.79, 46.87, and 0.9973, respectively [46]. Behnood et al. [47] evaluated the influence of several climate-related elements and the size of the population on the spread of COVID-19 by integrating the viral optimization algorithm (VOA) and ANFIS. They showed that the density of the population had a surprising impact on how well their constructed scenarios operated, highlighting the critical role that social distance plays in reducing the rate as well as the spread of COVID-19. They reported the RMSE as 22.47, MAE as 7.33, and $R^{2}$ as 0.83 [47].

Aora et al. [48] employed RNN-related LSTM variations to predict the number of positive patients in India. The LSTM model was chosen for forecasting daily as well as weekly COVID-19 patients with approximated errors of three percent for daily cases and eight percent for weekly cases based on the lowest false alarm rate. Depending on the volume of confirmed patients and everyday progression of the designation of COVID-19 hotspots, they divided Indian states into various zones [48]. A bidirectional LSTM network was used by Fokas et al. [49] to produce a reliable generalization of RNNs. This technique was used to forecast new COVID-19 infected individuals in the United States, Spain, Italy, Germany, France, and Sweden [49].

The regression model proposed by Yadav et al. [50] for the forecasting of COVID-19 cases was based on six regression analyses including quadratic, third-degree, fourth-degree, fifth-degree, sixth-degree, and exponential polynomials. The sixth-degree polynomial regression method was the best model for the forecasting of short-term new cases [50]. Geographical hierarchies were employed by Kim et al. [51] to develop Hi-COVIDNet in accordance with a neural network of two-level machinery based on information gathered from the continent and at the country level. This approach comprehended the complex connections between far-off nations and connected their unique risks of infection to the targeted community [51].

Three hybrid techniques for COVID-19 time-series forecasting were developed by Abbasimehr and Paki [52] by combining the Bayesian optimization algorithm with the multi-head attention, LSTM, and CNN deep learning techniques. These findings revealed that deep neural networks outperformed the benchmark model in terms of both the short-term and long-term predictions. In addition, the best deep learning model’s average SMAPE had short-term forecasts of 0.25 and long-term forecasts of 2.59 [52]. Additionally, deep neural networks (DNNs) have been proposed as a technique for prediction. This approach is a significant substitute for estimating a partial differential equation’s solution [11]. Based on the distribution of COVID-19 over three time periods, a recent work employed the K-means approach to group countries into various clusters [11].

## 4. Methods

**Figure 1:**
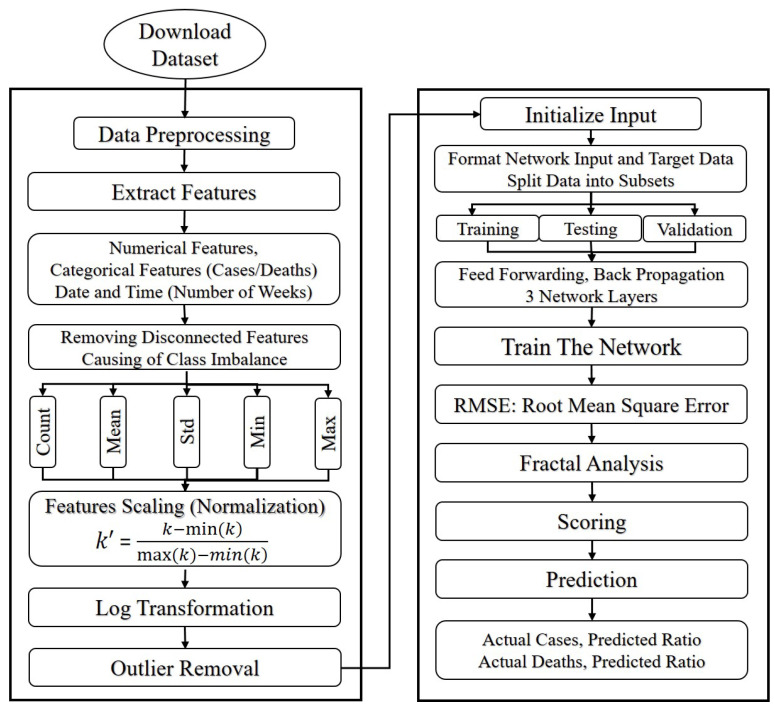
Flowchart diagram of our proposed model.

The proposed model for this work is the multi-layer perceptron neural network (MLPNN), whose flowchart/structure is illustrated in Figure 1. For this study, we collected data from the website of the World Health Organization [53]. The data used for this research were statistical data and contained no personally identifiable human photos, audios, videos, or other materials. Additionally, all procedures were conducted in accordance with the necessary rules and laws. As shown in Figure 1, the downloaded dataset was pre-processed using features extraction. We considered the categorical features (infected cases, number of deaths, and number of weeks) for this study. We tuned the model by removing the disconnected features that were causing the class imbalance, for example, we did not consider patients who had other diseases such as heart disease, cancer, diabetes, old age, etc. These features were causing a class imbalance, e.g., it was not necessary for all COVID-19 infected patients to be heart patients and vice versa. After removing the disconnected features, we normalized the data and initialized the input data by splitting it into subsets, i.e., 80% for training and 20% for testing. This splitting is typically made in a layered or randomized way to ensure the data are dispersed in the sample data of the subgroups, which minimizes biases or deviations in the data. The classification model that we utilized in the approach was trained using the training data and test data to evaluate the classifier’s performance over an unobserved subset of the data. We applied a three-layered feed-forward network (multi-layer perceptron neural network) model for training, testing, and validation. The MPLN is discussed briefly in the following sections.

### 4.1. Multi-Layer Perceptron Neural Network

**Figure 2:**
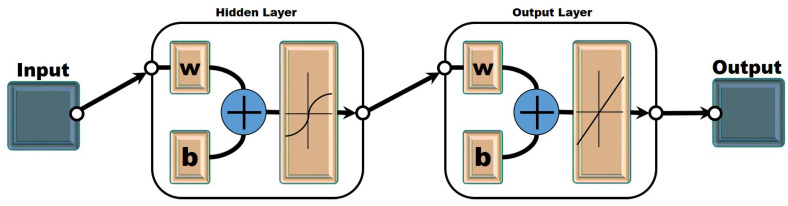
Architecture of Artificial Neural Network.

We employed a multi-layer perceptron neural network [54] and a feed-forward neural network with an input layer, hidden layers, and an output layer (see Figure 2). In this research, two separate multi-layer perceptron neural networks were trained, i.e., one for each of the goals— infected cases and deaths. The data of the infected cases and deaths were used from various countries including China, Bangladesh, Germany, Italy, India, Iran, Pakistan, and the United Kingdom.

Ten hidden neurons were used in a single hidden layer and a sigmoid function was also used. The sigmoid is the activation function, which is specified as

(1) $$N_{i}=\frac{1}{1+e^{-\sum w_{ki}}}$$

where $w_{ki}$ are the weights of input values and $N_{i}$ is the value of the hidden neurons.

In the output layer, there are two input neurons that show the number of deaths and number of active cases. Furthermore, Equation (2) defines the output of a hyperbolic tangent transfer function that ranges from −1 to +1, that is,

(2) $${\bar{N}}_{j}=\left(\frac{2}{1+e^{-2\sum w_{ij}}}\right)-1$$

where $w_{ij}$ is a weighted output between the hidden neuron *i* and the output neuron *j*. ${\bar{N}}_{j}$ is the output of *j*.

**Figure 3:**
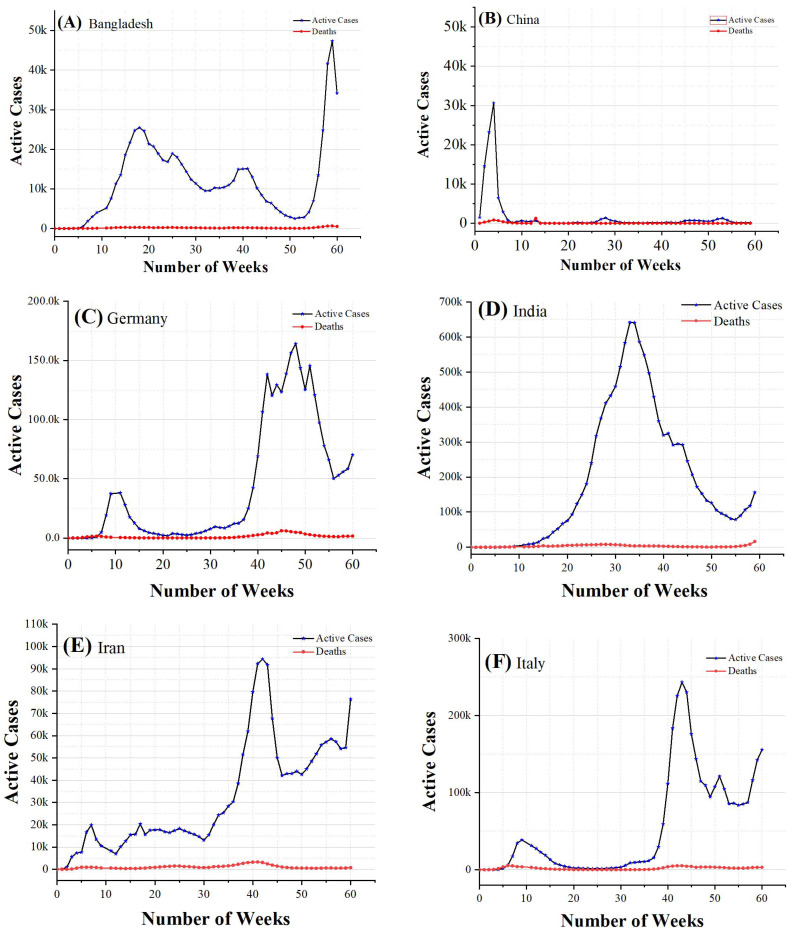

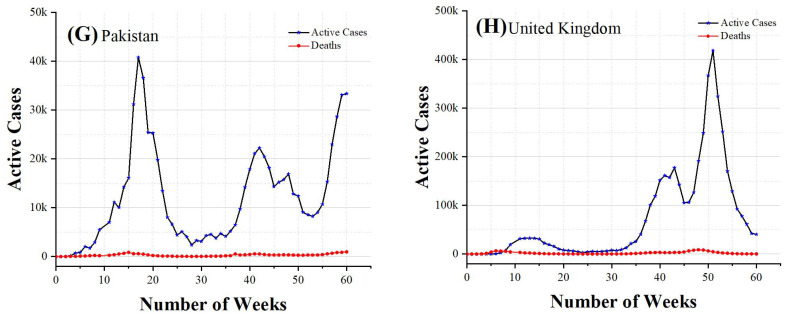
COVID-19 active cases and deaths in 60 weeks.

The best technique for calculating the best values for all the neural network variables, for example, the input and output weights, are used in the supervised learning approach. As a result, establishing the parameters of an ANN results in the development of an ANN model. Training through observed values and optimization is known as supervised learning (see Figure 3).

### 4.2. Mortality/Fatality Rate

The seriousness of a pandemic can be inferred from the fatality (case fatality ratio) rates/ratios, defined by

(3) $$CFR=\left(\frac{Deaths}{Confirmed\;cases}\right)100$$

where CFR is the case fatality ratio.

### 4.3. Cronbach’s Alpha

Cronbach’s alpha is a risk-adjusted evaluation metric that shows us how much the expected case returns differ from the actual case returns and whether deaths from COVID-19 are above or below the active cases/deaths. We calculated the actual cases and death ratio using Cronbach’s formula [55] (Equation (4)) as follows;

(4) $$C\alpha =\left(\frac{K}{K-1}\right)\left(\frac{S_{y}^{2}-\sum S_{i}^{2}}{S_{y}^{2}}\right)$$

where Cα denotes the actual cases and deaths, $S^{2}$ describes the number of samples, $S_{y}^{2}$ represents the variance in the total score. $S_{i}^{2}$ is the variance of the individual week, whereas $\sum S_{i}^{2}$ is the sum of the scores of the individual week.

### 4.4. Mean Absolute Error (MAE)

We used the mean absolute error (MAE) (see Equation (4)) to achieve forecasting with minimized errors. Based on the MAE’s values, the mean absolute scaled error (MASE) (Equation (5)) was calculated for the actual infected cases/deaths and predicted cases/deaths for future weeks.

(5) $$MAE=\frac{1}{k}\sum_{y=1}^{k}|e_{y}|$$

where y≤k and then the yth error $e_{y}$ is denoted by $e_{y}=x_{y-}\hat{x_{y}}$

### 4.5. Mean Absolute Scaled Error (MASE)

We computed the MASE (mean absolute scaled error) using the actual numbers of infected cases and deaths and the forecasted values of the cases and deaths using the following equation (Equation (6)).

(6) $$MASE=\frac{\frac{1}{k}\sum_{y=1}^{k}\left|e_{y}\right|}{\frac{1}{k-1}\sum_{y=2}^{k}\left|x_{y}-x_{y-1}\right|}$$

### 4.6. Symmetric Mean Absolute Percentage Error (SMAPE)

We further calculated our data using SMAPE (Equation (7)). SMAPE uses the squared values such as the root mean square error (RMSE) (Equation (8)).

(7) $$SMAPE=\frac{1}{k}\sum_{y=1}^{k}\frac{\left|e_{y}\right|}{\left(\left|x_{y}\right|+\left|{\hat{x}}_{y}\right|\right)/2}$$

where *k* represents the sample size, $x_{y}$ indicates the actual values of the infected cases/deaths, and ${\hat{x}}_{y}$ indicates the forecasted values of the cases/deaths. y≤k and then the yth error $e_{y}$ is denoted by $e_{y}=x_{y-}\hat{x_{y}}$

### 4.7. Root Mean Square Error (RMSE)

The RMSE computes the difference of the error between two actual values and the forecasted values. We compared the anticipated value and real measurements, i.e., (a) the predicted values and (b) the observed values, respectively. We divided the total number of observations by the sum of all the values. Finally, we calculated the root mean square error (RMSE) (8) below:

(8) $$RMSE=\sqrt{\frac{{\left(O-E\right)}^{2}}{n}}.$$

where *n* represents the total number of infected people, *O* denotes the number of observed values of actual cases, and *E* represents the number of the total expected values.

### 4.8. Data Pre-Processing and Experimental Setup

Authentic sources [53] were used to collect the data. We used the datasets of various countries including China, Bangladesh, Germany, Italy, India, Iran, Pakistan, and the United Kingdom. This study contains no personally identifiable human photos, audios, videos, or other materials. All procedures were followed in compliance with the necessary rules and regulations. A Windows 10, 64-bit operating system, with 16 GB of RAM was employed. For the training and validation datasets, we used CSV files. We normalized the data and initialized the input data by splitting them into subsets, i.e., 80% for training and 20% for testing. This splitting is typically made in a layered or randomized way to ensure the data are dispersed in the sample data of the subgroups, which minimizes biases or deviations in the data. K-fold validation was used to validate the performance of our proposed framework.

### 4.9. Model Forecasting

A time-series analysis is a very important component of deep learning and is utilized for forecasting. Time is the only input variable (independent feature) used to forecast the target feature (dependent feature) in time-series data, which are a type of univariate regressive data. It is used to predict the future values of coming occurrences and is crucial for predicting the occurrence of respiratory disorders such as COVID-19. Positive cases are growing every day, thus it is important to predict whether the rate of growth will continue based on earlier data. Governments can mobilize resources to prevent disease transmission based on forecasts and take action in the future to slow the pace of infection increase without impacting more citizens. Forecast numbers cannot be assured because predictions depend entirely on past patterns. To counter a pandemic emergency such as COVID-19, governments can use this approximate projection of occurrences to evaluate future resource management. This section discusses the actual situation with COVID-19-infected cases and forecasts future situations for infected cases and deaths.

Table 4 exhibits the CFRs for the selected countries as well as globally. A CFR of 5.33% was reported for China, 2.99% for Italy, 2.85% for the United Kingdom, 1.17% for India, 1.58% for Bangladesh, and 2.25% for Pakistan, whereas a global CFR of 2.08% was reported [53]. Due to the large number of deaths at the beginning of the pandemic, China had the highest CFR among the other countries; however, after May, China’s fatalities decreased as a result of the lockdowns used to contain the pandemic. It is worth noting that the CFR is influenced by the number of tests performed and the size of the population. Therefore, a solid approach should be developed to avoid this constraint. The CFR changes when new cases of infection and fatalities appear. Table 5 and Table 6 show the results for the alpha, MASE, SMAPE, MAE, and RMSE for actual cases and deaths, respectively. Alpha returned a base value parameter of between 0 and 1. MASE returned a mean absolute scaled error measurement of the forecasting. The symmetric mean absolute percentage measurement parameter was returned by the SMAPE function. The MAE returned the mean absolute error and the RMSE returned the root mean squared error metric. Figure 3 denotes a detailed visualization of the weeks, that is, 60 weeks on the x-axis and the number of infected patients plus the number of deaths on the y-axis. Graph (A) shows the data from Bangladesh, graph (B) from China, graph (C) from Germany, graph (D) from India, graph (E) from Iran, graph (F) from Italy, graph (G) from Pakistan, and graph (H) shows the data from the United Kingdom.

Table 7 shows the test results of the best models for the death forecasting. Table 8 shows the weekly death forecasts for the upcoming months. The model forecast results for India show an increase in weekly deaths at a faster rate compared to the other specified countries. Consequently, if the same strategy is maintained, COVID-19 will be completely out of control in India and fatalities could reach more than 121 thousand by the start of the upcoming year. The weekly death forecasts for Pakistan, Bangladesh, and Iran show decreases but at a relatively slow rate. The forecasts indicate that for Pakistan, COVID-19 deaths in the 1st week of the upcoming month in 2022 are 380, and this number will not exceed 537, with a confidence level of 95%. However, weekly deaths will reduce to 316, indicating a reasonably considerable difference in a couple of months. For Iran, the forecast for deaths is 1367 and will not exceed 1732, whereas for Bangladesh, it is 198 and will not exceed 292. The forecasting results for Germany are also declining at a slower rate. The forecast results show that in the last week of the first month, the weekly deaths will be 775 and will not exceed 5812, with a confidence level of 95%. The upper limit suggests an alarming situation. It is highly recommended for their governments to take steps and implement new policies as preventive measures regarding the pandemic situation. The forecast for the UK shows that weekly deaths will increase and in the last week of the upcoming month will be 126 and not exceed 8876. The results indicate that these countries’ current strategies are working effectively in controlling the pandemic but the future situation may worsen, as shown by the upper limit of the forecast; it is highly recommended that they revise their policies in a timely manner.

Finally, regarding Italy’s future scenario, the situation will not be as difficult as in India. However, there is a considerably high weekly deaths forecast (more than a couple of hundred) for the end of the current year and the start of the next year. Table 7 gives a brief overview of the best models’ test results for death forecasting. The WHO should give special consideration and help countries, such as India, Italy, and others with high mortality forecasts for COVID-19, to fight against the pandemic.

**Figure 4:**
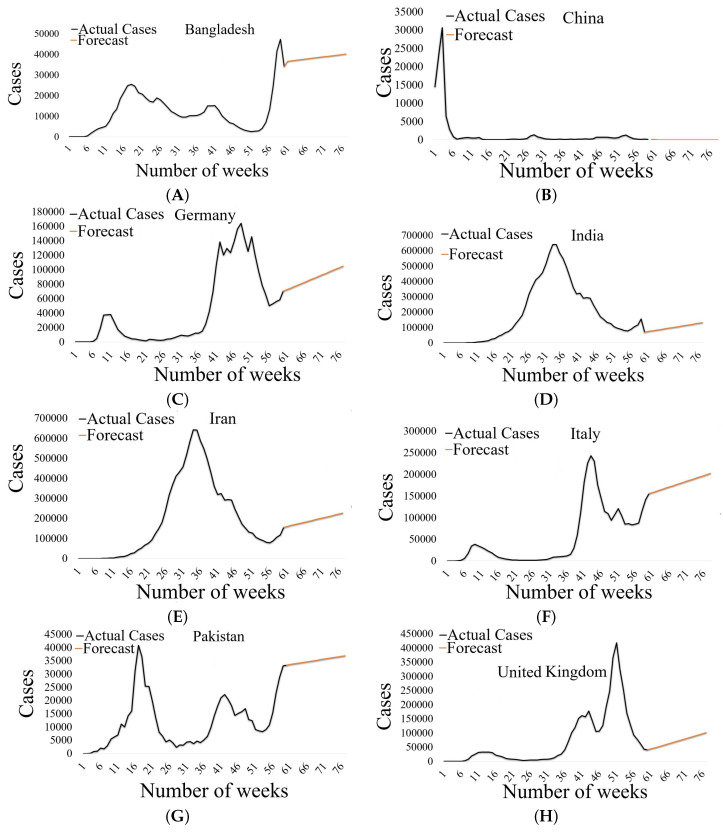
Actual cases in 60 weeks and predicted cases in future weeks.

Figure 4 represents a detailed view of the number of weeks on the x-axis and the number of actual cases and predicted cases on the y-axis. Graph (A) shows the data from Bangladesh, graph (B) from China, graph (C) from Germany, graph (D) from India, graph (E) from Iran, graph (F) from Italy, graph (G) from Pakistan, and graph (H) shows the data from the United Kingdom.

**Figure 5:**
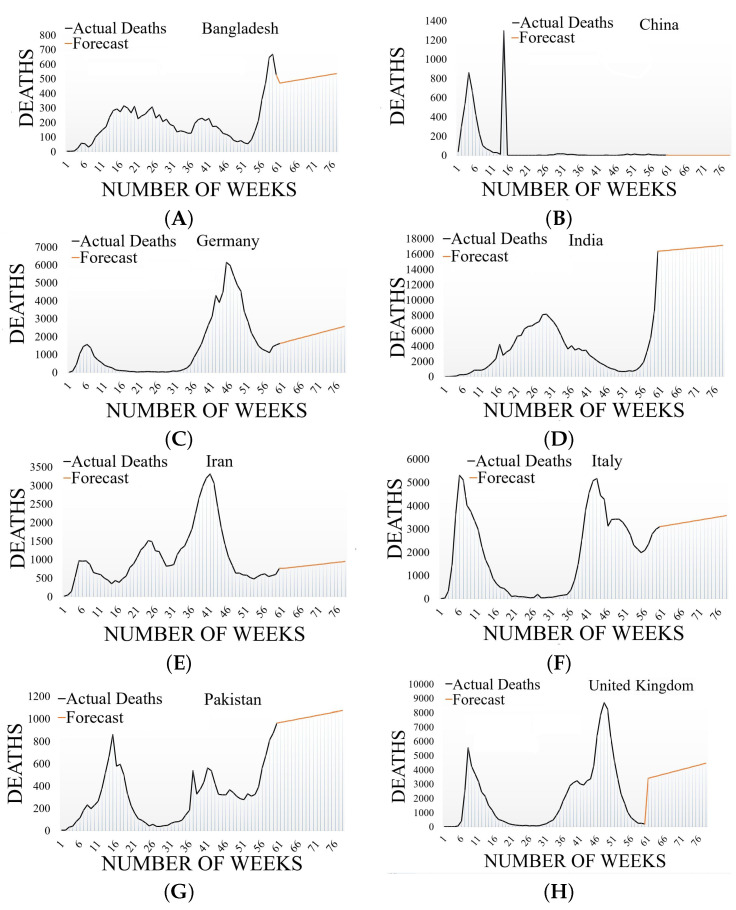
Actual deaths in 60 weeks and predicted deaths in future weeks.

Figure 5 shows a detailed view of the total number of weeks on the x-axis and the number of actual deaths and predicted deaths on the y-axis. Graph (A) shows the data from Bangladesh, graph (B) from China, graph (C) from Germany, graph (D) from India, graph (E) from Iran, graph (F) from Italy, graph (G) from Pakistan, and graph (H) shows the data from the United Kingdom.

### 4.10. The Model’s Performance

**Figure 6:**
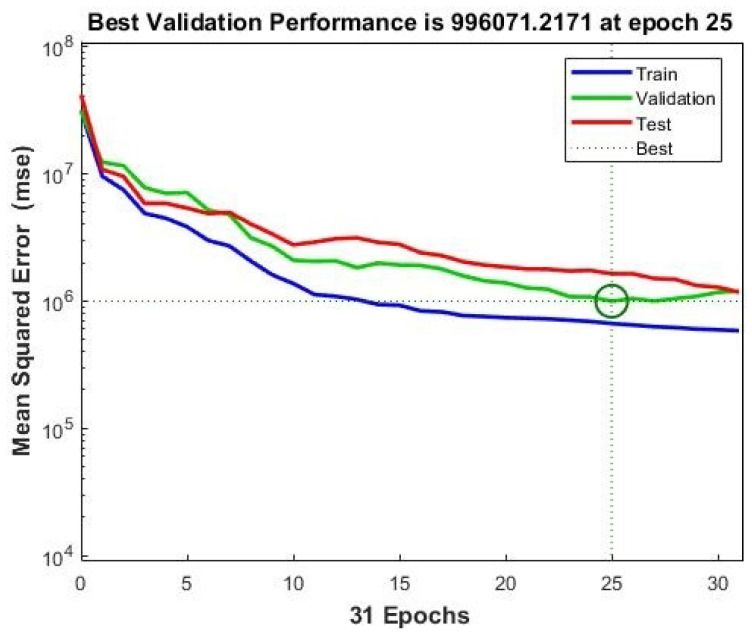
Training, validation, test, and best observed results.

The results of the best accuracy, training, testing, and validation of our framework are briefly summarized in Figure 6. The results show a 99.60% accuracy, which means that the validation effectiveness is satisfactory. These outcomes were seen when initializing the input parameters for the model, indicating that the model was properly trained and the data were error-free.

**Figure 7:**
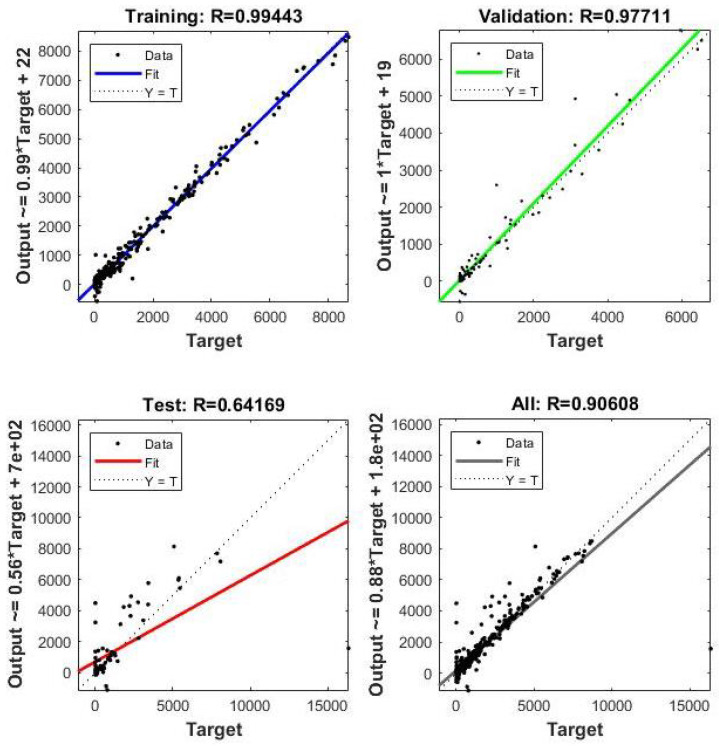
Best fitting for training, validation, testing, and all observed results.

Figure 7 gives a brief visualization of the output results. The value of the training correlation coefficient of the target output was observed to be 99.44%, the validation was observed to be 99.77%, the testing was observed to be 64.16, and the overall value was observed to be 90.6%, which means that our model was efficient. The correlation quantifies the strength of a linear relationship between two variables. We used a correlation to investigate whether a relationship existed between the variables to assume or fit a specific model to our data. A value close to 1 (90.6% in this research) indicated that there was a positive linear relationship between the data columns, which means that our proposed model was precisely or accurately working on the given dataset.

## 5. Challenges and Future Directions

We discuss the current issues associated with utilizing Artificial Intelligence methods to resolve the COVID-19 pandemic. Furthermore, we demonstrate how machine learning and deep learning can assist in preventing the transmission rate of COVID-19 in the future.

### 5.1. Challenges

Applications based on AI for investigating COVID-19 are presently facing numerous hurdles, for example, the scarcity, legislation, and inaccessibility of substantial data; there are a lot of noisy data as well as false feedback; the inadequate alertness of the juncture between medicine and computer science; the issue of security and data privacy, etc.

**Policies and Regulations**

As the epidemic spreads and the numbers of reported affected and deceased people rises, several measures to limit the outbreak have been discussed, for example, social distancing and lockdowns. Authorities have an important role in establishing regulations and rules to motivate citizens, experts, educators, entrepreneurs, medical centers, technology giants, and large corporations to cooperate in COVID-19 mitigation during an outbreak.

**Large-scale training data are scarce and unavailable**

Many Artificial Intelligence deep learning (AIDL) systems rely on large-scale datasets, including diagnostic image processing, with a variety of environmental variables. Yet, because of COVID-19’s explosive expansion, there are inadequate resources for AI. In practice, analyzing datasets is a time-consuming task and demands the support of trained health professionals.

**Noisy data and speculation on the internet**

The problems occur as a result of a reliance on easily available online social networking sites; vast amounts of audio/video, fake information, and misleading news have been reported in thousands of different online channels without any substantial modifications. Artificial intelligence-based techniques appeared to be slow when evaluating and processing noisy data. Furthermore, the outputs of Artificial Intelligence ML and DL techniques become skewed with noisy data. These issues reduce the performance and efficiency of Artificial intelligence algorithms, especially for epidemic forecasts and spreading analyses.

**Lack of integration between computer science and medicine arenas**

Numerous Artificial Intelligence experts have a strong hold on computer science applications, but considerable expertise in diagnostic imaging, epidemiology, pharmacology, and other relevant domains is also required to incorporate other medical information into artificial intelligence methods in the war against COVID-19. To handle COVID-19, it will be essential to arrange for specialists from different majors to work together and integrate data from numerous works.

**Data security and privacy**

In the era of Artificial Intelligence, the cost of acquiring confidentiality of data is incredibly low. In the presence of healthcare issues such as the current pandemic situation, several government agencies strove to gather a wide range of personal data including contact numbers, ID numbers, and medical data. How to properly maintain individual confidentiality and human rights during Artificial Intelligence discovery and handling is a topic worth tackling.

**Unstructured data or incorrect structural data (e.g., numerical, text, and image data)**

Working with incorrect facts and ambiguous data in textual material can be challenging. It is possible for large amounts of data from several sources to be erroneous. Furthermore, a lot of data makes it difficult to extract valuable bits of metadata.

**Early detection of COVID-19 via image analysis such as chest X-rays and CT scans**

Handling unbalanced datasets results in insufficient diagnostic imaging and extensive training periods and being unable to describe the problems of the efficient outcomes.

**Risk assessments of old-age people and patients with other diseases**

Old-age people should be screened, functioning treatments and cures should be discovered, risk assessments should be conducted, survival projections should be made, healthcare should be provided, and medical source planning should be conducted. The task at hand is to obtain the physical features and therapeutic outcomes for patients. An additional challenge is dealing with low-quality data, which can lead to skewed and incorrect predictions for old-age people and people with other diseases, for example, heart disease, diabetes, asthma, and so on.

### 5.2. Future Research Direction

Artificial Intelligence and blockchain-based solutions can also contribute to the fighting the outbreak in the following ways.

**Non-contact illness diagnostics**

Using automatic feature categorization in X-ray and CT imaging during COVID-19 outbreaks will successfully limit the outbreaks. A patient’s posture can be detected and CT image detection, X-rays, and smart camera facilities can all be utilized in AI-based systems.

**Video diagnostics and consulting remotely**

To deliver COVID-19 hospital admissions and early diagnosis data, a mix of Artificial Intelligence and natural language processing (NLP) modules can be utilized to construct remote diagnostic programs and automation systems.

**Bio-technological research**

AI-based algorithms can be utilized to accurately examine biomedical knowledge in terms of biotechnological research, such as major protein structures, genomic sequencing, and viral itineraries, to determine protein compositions and viral components.

**Vaccination and drug development**

AI-based algorithms can be used to find prospective medications and vaccinations, as well as replicate drug–protein and vaccine–receptor pairings, allowing for the prediction of future drug and vaccine responses in COVID-19 patients.

**Fake information must be identified and screened**

In order to provide real, accurate, and comprehensive COVID-19 statistics, Artificial Intelligence models must be used to filter out erroneous news and material online. Blockchain-based [56] systems can be used to track and trace the actual information source.

**Impact analysis and appraisal**

Various sorts of computations can use machine learning, deep learning techniques to evaluate the influence of different social management strategies on the spread of the pandemic. Data could then be used to evaluate logical and efficient strategies for disease prevention and control in the general public.

**Tracking of patients’ contacts**

By establishing social networking sites and an information architecture, blockchain-based federated learning can be used to detect and track the characteristics of individuals residing in close proximity to COVID-19 sufferers, effectively anticipating and tracking the pandemic progression.

**Smart robots**

Robotic systems are likely to be used in activities, for example, public sanitation, deliveries, supply chains, and in healthcare infrastructures that do not require human resource management, e.g., medical treatment. This can stop the COVID-19 virus from spreading.

**Future work with descriptive federated learning methods**

The effectiveness of federated learning methods and graphic properties that cause distinctions between COVID-19 and other strains of tuberculosis must be determined. This will aid radiologists and doctors in being more conscious of the infection and effectively analyzing probable COVID-19 X-rays and CT imaging data.

**Importance of COVID-19 diagnostic tools and treatment**

These are both necessary but the early detection of COVID-19 is far more important. Substantial future study efforts based on ML and DL are needed in order to identify COVID-19 therapies.

## 6. Conclusions

The applications of operational research that uses mathematical, statistical, and demographic modeling are crucial in assisting decision makers in education, health, socioeconomic, and other aspects of daily life. By adopting preventative measures beforehand, the transmission of COVID-19 could be considerably slowed. In order to maintain attention on the most sensitive location, country, or region, scientists, research professionals, and global leaders must be informed in advance of the emergency scenarios. For forecasting the pandemic situation, this study proposed a multi-layer perceptron neural network (MLPNN) with the integration of Cronbach’s alpha and the MAE, MASE, SMAPE, RMSE, and CFR. We also focused on the current challenges in preventing the outbreak from further spread and what is needed in the future to normalize social and economic activities. High accuracy was observed in estimating the percentages of afflicted patients and deaths. According to the MLPNN model’s encouraging results, the volume of COVID-19 people in India will rise in the upcoming weeks and the death rate will also rise. This was evident from the 95% upper limit confidence interval, which was becoming wider for subsequent weeks. In general, forecasts for the near future were more precise compared to the longer term. Furthermore, providing the breakdown of the forecasting for each of the past COVID-19 variants could be a very interesting contribution to the research and will be explored in future studies. For this research, we could not find actual data about the numbers of patients who were affected by the particular variants in the selected countries.

## Figures and Tables

**Table 1:** Some of the recent variants categorized by WHO.

Scientific Name	Name Given by the WHO	Spike Protein Substitutions	Attributes
		70del, A570D,	1. 50% higher spread capability
B..1.1.7	Alpha	69del,	2. Possible enhanced severity based
		(S494P),	on hospital admissions and case
		(E484K),	mortality rates
		P681H, 144del,	3. Treatment with EUA monoclonal
		N501Y, D614G,	antibodies has no effect on
		T716I, D1118H,	susceptibility
		S982A	4. Minimal effect on recovery and
		(K1191N)	post-vaccination serum
			neutralizing
		A701V, D215G,	1. 50% higher spread capability
B.1.351	Beta	D614G, D80A,	2. Susceptibility to a combination
		E484K,	of bamlanivimab and etesevimab
		N501Y,	monoclonal antibody treatment
		K417N,	was drastically lowered; however,
		241del	there are other EUA monoclonal
		242del	antibody treatments available
		243del	3. Condensed neutralization by
			convalescent and post-vaccination sera
		D138Y, D614G,	1. Susceptibility to the combination
P.1	Gamma	E484K, H655Y,	of bamlanivimab and etesevimab
		K417T, L18F,	monoclonal antibody treatment was
		N501Y, P26S	drastically lowered; however, there
		R190S, T20N,	are other EUA monoclonal antibody
		T1027I	treatments available
			2. Condensed neutralization by convalescent
			and post-vaccination sera
		T95I, G142D,	1. Higher spread capability
B.1.617.2	Delta	T19R, (V70F),	2. Possible decrease in neutralization
		R158G, (A222V),	by some EUA monoclonal antibody
		E156-, F157-,	treatments
		D614G, D950N,	3. Possible decrease in neutralization
		(W258L), (K417N)	by post-vaccination sera
		P681R, L452R,	
		T478K	

**Table 2:** Variants of Interest (VOI) [27].

Labeled by the WHO	Additional Variations in the Lineage	Country First Discovered	Spike Changes of Interest	Date of First Detection	Influence on Transmissibility	Possibility of a Negative Effect on Immunity	Transmission in Europe
Eta	E484K	Nigeria	Q677H	December 2020	–	Neutralization (m) [33]	Communities
			D614G				
			B.1.525				
Epsilon	B.1.429,	United States	D614G	September 2020	Ambiguous [26]	Neutralization (v) [26]	Inconsistent/Travels
	B.1.427		L452R				
Theta	P.3	Philippine	D614G	January 2021	Yes (m) [34]	Neutralization (m) [33]	Inconsistent/Travels
			E484K				
			P681H				
			N501Y				
	B.1.616	France	D614G	February 2021	Recognition (c) [25]	–	One-Time Occurrence
			G669S				
			H655Y				
			V483A				
Kappa	B.1.617.1	India	D614G	December 2020	Yes (v) [35]	Neutralization (v) [25,36]	Multiple Occurrences
			E484Q				
			L452R				
			P681R				
	B.1.620	Not clear	D614G	February 2021		Neutralization (m) [33,37]	Multiple Occurrences
			E484K				
			P681H				
			S477N				
	B.1.621	Colombia	D614G	January 2021	Yes (m) [34]	Neutralization (m) [33]	Inconsistent/Travels
			E484K				
			P681H				
			N501Y				
			R346K				

**Table 3:** Variants under observation [27].

Labeled by the WHO	Additional Variations in the Lineage	Country First Discovered	Spike Changes of Interests	Date of First Detection	Influence on Transmitability	Possibility of a Negative Effect on Immunity	Proof of Link to Intensity	Transmission in Europe
	B.1.617.3	India	D614G	February 2021	Yes (m) [34]	Neutralization (m) [26,33]	–	Not found
			E484Q					
			L452R					
			P681R					
	B.1.214.2	not clear (b)	D614G	December 2020	–	–	–	found (a)
			ins214TDR					
			N450K					
			Q414K					
	A.23.1+E484K	UK	E484K	December 2020	–	Neutralization (m) [33]	–	found (a)
			Q613H					
			V367F					
	A.27	not clear (b)	A653V	December 2020	Yes (m) [34]	Neutralization (m) [26]	–	found (a)
			N501Y					
			L452R					
			H655Y					
	A.28	not clear (b)	E484K	October 2020	–	Neutralization (m) [33]	–	found (a)
			H655Y					
			N501T					
	C.16	not clear (b)	L452R	December 2020	–	Neutralization (m) [33]	–	found (a)
			D614G					
Labmda	C.37	Peru	D614G	December 2020	–	–	–	found (a)
			F490S					
			L452Q					
	B.1.351+P384L	South Africa	A701V	December 2020	Yes (v) [39]	Escape (v) [40,41]	not clear [42]	found (a)
			D614G					
			E484K					
			K417N					
			N501Y					
			P384L					
	B.1.351+E516Q	not clear (b)	A701V	January 2021	Yes (v) [39]	Escape (v) [40,41]	not clear [42]	found (a)
			D614G					
			E484K					
			E516Q					
			K417N					
			N501Y					
	B.1.1.7+L452R	UK	D614G	January 2021	Yes (v) [34]	Neutralization (m) [26]	Yes (v) [43]	found (a)
			L452R					
			P681H					
			N501Y					
	B.1.1.7+S494P	UK	D614G	January 2021	Yes (v) [34]	Neutralization (m) [36]	Yes (v) [43]	found (a)
			N501Y					
			P681H					
			S494P					
	C.36+L452R	Egypt	D614G	December 2020	–	Neutralization (m) [26]	–	found (a)
			L452R					
			Q677H					
	AT.1	Russia	D614G	January 2021	–	Neutralization (m) [33]	–	found (a)
			E484K					
			ins679GIAL					
			N679K					
Iota	B.1.526	US	A701V	December 2020	–	Neutralization (m) [33]	–	found (a)
			D614G					
			E484K					
	B.1.526.1	US	D614G	October 2020	–	Neutralization (m) [26]	–	found (a)
			L452R					
	B.1.526.2	US	D614G	December 2020	–	–	–	found (a)
			S477N					
	B.1.1.318	not clear (b)	D614G	January 2021	–	Neutralization (m) [33]	–	found (a)
			E484K					
			P681H					
Zeta	P.2	Brazil	D614G	January 2021	–	Neutralization (m) [33]	–	found (a)
			E484K					
	B.1.1.519	Mexico	D614G	November 2020	–	Neutralization (m) [26]	–	found (a)
			T478K					
	AV.1	UK	D614G	March 2021	–	Neutralization (m) [33]	–	found (a)
			E484K					
			P681H					
			N439K					
	P.1+P681H	Italy	D614G	February 2021	–	not clear	–	–
			H655Y					
			E484K					
			N501Y					
			P681H					
			K417T					

**Table 4:** Case Fatality Ratios of COVID-19 [53].

Countries	CFR (%)
Bangladesh	1.58
India	1.17
China	5.33
Pakistan	2.25
Iran	2.75
Germany	2.39
Italy	2.99
United Kingdom	2.85
World-wide	2.08

**Table 5:** Forecasting metric results for all active cases.

Country	Alpha	MASE	SMAPE	MAE	RMSE
Bangladesh	0.5	5	0.47	66,660.96	10,128.56
China	0.9	0.15	0.45	205.99	270.5
Germany	0.25	3.2	0.34	19,705.31	22,740.67
India	1	0.86	0.2	20,950.76	30,570.1
Iran	1	0.59	0.14	14,199.15	18,578.85
Italy	1	1.33	0.12	12,815.17	15,552.92
Pakistan	1	0.97	0.17	2418.55	2875.24
United Kingdom	0.1	15.74	0.69	135,707.55	152,719.52

**Table 6:** Forecasting metrics results of deaths.

Country	Alpha	MASE	SMAPE	MAE	RMSE
Bangladesh	0.25	5.3	0.58	137.17	180.56
China	0.25	0.23	2	21.05	21.6
Germany	1	0.88	0.13	213.27	313.1
India	0.1	6.04	0.8	2508.59	4225.44
Iran	0.9	0.28	0.09	52.28	64.89
Italy	1	0.45	0.07	176.9	199.38
Pakistan	0.5	0.9	0.13	58.35	71.94
United Kingdom	0	5.84	1.04	2523.59	2861.2

**Table 7:** Test results of the best models for death forecasting.

Countries	Best Model	MAPE	DFT *p*-Value *	ACF1 **
Bangladesh	1,1,0	19.22 *	0.04	−0.002
China	5,1,1	inf *	0.01	−0.077
Germany	1,1,0	24.87	0.01	−0.042
India	0,2,0	16.24 *	0.01	0.089
Iran	0,1,3	13.39 *	0.01	0.040
Italy	4,1,0	36.17	0.01	0.010
Pakistan	1,1,0	18.62 *	0.01	−0.078
UK	2,1,1	27.59	0.01	0.029
World	1,1,0	12.01	0.05	−0.073

* Duckey Fuller test, alternative hypothesis: stationary; ** First-order autocorrelation function.

**Table 8:** Upcoming 16-week forecast for weekly deaths from COVID-19 for Bangladesh, China, Germany, India, Pakistan, Iran, Italy, the United Kingdom, and the world.

		Pakistan		Iran	
Month	Week	Point Forecast	95% CI(Upper)	Point Forecast	95% CI(Upper)
1	1	380	537	1367	1732
	2	342	615	1362	2070
	3	326	696	1338	2493
	4	320	772	1338	2878
2	1	317	842	1338	3185
	2	316	905	1338	3447
	3	316	962	1338	3681
	4	315	1016	1338	3893
3	1	315	1065	1338	4089
	2	315	1112	1338	4271
	3	315	1156	1338	4443
	4	315	1198	1338	4606
4	1	315	1238	1338	4762
	2	315	1276	1338	4910
	3	315	1313	1338	5053
	4	315	1349	1338	5190
		**India**		**Italy**	
Month	Week	Point Forecast	95% CI(Upper)	Point Forecast	95% CI(Upper)
1	1	30,506	32,639	714	1522
	2	31,094	35,864	697	2310
	3	31,682	39,664	740	3164
	4	32,270	43,954	798	3910
2	1	32,858	48,679	845	4500
	2	33,446	53,796	871	4944
	3	34,034	59,275	878	5279
	4	34,622	65,091	872	5549
3	1	35,210	71,223	861	5787
	2	35,798	77,655	851	6014
	3	36,386	84,372	845	6241
	4	36,974	91,361	843	6469
4	1	37,562	98,612	844	6695
	2	38,150	106,113	846	6917
	3	38,738	113,858	848	7132
	4	39,326	121,836	849	7338
		**Bangladesh**		**UK**	
Month	Week	Point Forecast	95% CI(Upper)	Point Forecast	95% CI(Upper)
1	1	198	292	92	978
	2	194	360	122	2148
	3	192	420	140	3333
	4	191	473	145	4344
2	1	191	520	141	5122
	2	190	562	133	5695
	3	190	599	126	6126
	4	190	634	122	6476
3	1	190	667	120	6790
	2	190	697	121	7094
	3	190	726	123	7400
	4	190	753	125	7709
4	1	190	779	126	8015
	2	190	804	126	8314
	3	190	828	126	8601
	4	190	851	126	8876
		**Germany**		**China**	
Month	Week	Point Forecast	95% CI(Upper)	Point Forecast	95% CI(Upper)
1	1	881	1587	1	100
	2	824	2078	1	204
	3	798	2525	1	277
	4	786	2923	1	354
2	1	780	3278	1	391
	2	777	3597	0	400
	3	776	3887	0	404
	4	776	4154	0	404
3	1	775	4403	0	404
	2	775	4635	0	412
	3	775	4855	1	427
	4	775	5063	1	458
4	1	775	5262	1	497
	2	775	5453	1	522
	3	775	5636	1	539
	4	775	5812	0	545
**World**					
Month	Week	Point Forecast		95% CI(Upper)	
1	1	73,427		83,134	
	2	71,635		89,025	
	3	70,763		94,891	
	4	70,339		100,348	
2	1	70,133		105,326	
	2	70,032		109,863	
	3	69,983		114, 019	
	4	69,960		117,857	
3	1	69,948		121,430	
	2	69,942		124,779	
	3	69,940		127,941	
	4	69,938		130,941	
4	1	69,938		133,801	
	2	69,937		136,539	
	3	69,937		139,169	
	4	69,937		141,702	
